# Identification and Validation of Core Genes Involved in the Development of Papillary Thyroid Carcinoma via Bioinformatics Analysis

**DOI:** 10.1155/2019/5894926

**Published:** 2019-09-08

**Authors:** Xiaoyan Li, Jing He, Mingxia Zhou, Yun Cao, Yiting Jin, Qiang Zou

**Affiliations:** ^1^Department of General Surgery, Huashan Hospital, Fudan University, Shanghai, China; ^2^Department of Gastroenterology, Xinhua Hospital, School of Medicine, Shanghai Jiao Tong University, Shanghai, China

## Abstract

**Background:**

Papillary thyroid carcinoma (PTC) is a common endocrine malignant neoplasm, and its incidence increases continuously worldwide in the recent years. However, efficient clinical biomarkers were still deficient; the present research is aimed at exploring significant core genes of PTC.

**Methods:**

We integrated three cohorts to identify hub genes and pathways associated with PTC by comprehensive bioinformatics analysis. Expression profiles GSE33630, GSE35570, and GSE60542, including 114 PTC tissues and 126 normal tissues, were enrolled in this research. Gene ontology (GO) and Kyoto Encyclopedia of Genes and Genomes (KEGG) pathway enrichment analyses were utilized to search for the crucial biological behaviors and pathways involved in PTC carcinogenesis. Protein-protein interaction (PPI) network was constructed, and significant modules were deeply studied.

**Results:**

A total of 831 differentially expressed genes (DEGs) were discovered, comprising 410 upregulated and 421 downregulated genes in PTC tissues compared to normal thyroid tissues. PPI network analysis demonstrated the interactions between those DEGs, and top 10 pivotal genes (*TGFB1*, *CXCL8*, *LRRK2*, *CD44*, *CCND1*, *JUN*, *DCN*, *BCL2*, *ACACB*, and *CXCL12*) with highest degree of connectivity were extracted from the network and verified by TCGA dataset and RT-PCR experiment of PTC samples. Four of the hub genes (*CXCL8*, *DCN*, *BCL2*, and *ACACB*) were linked to the prognosis of PTC patients and considered as clinically relevant core genes via survival analysis.

**Conclusion:**

In conclusion, we propose a series of key genes associated with PTC development and these genes could serve as the diagnostic biomarkers or therapeutic targets in the future treatment for PTC.

## 1. Introduction

Thyroid cancer accounts for 96% malignant tumors of the head and neck, which is the most common malignant tumor in endocrine organs and the fifth most common cancer in women worldwide [1, 2]. The incidence of thyroid cancer continues to increase during the past 30 years, which has the fastest growth rate among all solid malignant tumors, generating a serious impact on the quality of life and huge social economic burden for patients [3]. The 2017 edition of American cancer statistics indicated that the incidence of thyroid cancer accounted for 3.4% of all malignant tumors. According to an analysis of thyroid cancer data from the World Health Organization during 1960 to 2012, the incidence of thyroid cancer in most countries and regions around the world presents a rapid growth in the last half century [4]. Thyroid cancer can be classified into four subtypes: papillary thyroid carcinoma (PTC), follicular thyroid carcinoma (FTC), medullary thyroid carcinoma (MTC), and anaplastic thyroid carcinoma (ATC), among which PTC is the most common type, accounting for about 80% of all thyroid cancer. Most of the patients with differentiated thyroid cancer (DTC) can obtain satisfactory therapeutic effects through surgical excision and radioactive iodine therapy and an overall 10-year survival rate exceeding 90% [5, 6]. However, a portion of thyroid cancer patients develop distant metastases and exhibit poor prognosis [7].

At present, the cause of PTC is not very clear. Several studies reported that the incidence of thyroid cancer is influenced by many factors, such as sex, age, region, and ethnic differences [8, 9]. Many studies have reported that the pathogenesis of PTC is related to BRAF mutation and RET/PTC and PAX8/PPAR rearrangement; one hypothesis speculated that the activation of serine/threonine kinase caused by mutations of BRAF at T1799A and V599E may be an alternative mechanism of oncogenic mitogen-activated protein kinase (MAPK) pathway activation [10–12], and other groups found that RET/PTC rearrangement promotes the proliferation and migration of PTC cells by regulating nuclear factor-*κ*B (NF-*κ*B) activity and relative oncogenic protein expression [13–15]. PAX8/PPAR rearrangement was also involved in thyroid tumorigenesis. PAX8 regulates thyroglobulin, thyroid peroxidase, and thyrotropin receptor gene promoters; it also regulates the differentiation and growth of thyroid cells [16]. PPAR*γ* is one of the nuclear receptors, which participates in inflammation, cell cycle control, apoptosis, and oncogenesis. PAX/PPAR rearrangement intervenes the transcription pathway, upregulates relative gene promoter activity, and finally promotes PTC progression [17–19]. However, these theories still cannot explain the pathogenesis of all PTCs. Therefore, further studies of the molecular mechanisms of PTC and seeking novel biomarkers for PTC seem to be of great significance and clinical value.

With wide application of high-throughput sequencing and public databases, the bioinformatics analysis has been broadly used as a powerful tool for cancer diagnosis, classification, and prognosis prediction, especially in obtaining gene general alterations during tumorigenesis. In this study, three original microarray datasets GSE33630, GSE35570, and GSE60542 were selected from Gene Expression Omnibus (GEO, https://www.ncbi.nlm.nih.gov/geo/) with a total of 114 PTC samples and 126 normal thyroid tissues, which owns the largest sample size compared with a previously published study with a total of 92 PTC samples and 79 normal thyroid tissue samples [20]. With the adhibition of the GEO2R online tool, 831 DEGs (differentially expressed genes) were detected: 410 of those genes were upregulated and 421 were downregulated. Based on those DEGs, the functional roles and related pathways were jointly performed by Database for Annotation, Visualization, and Integrated Discovery (DAVID) and other online tools. In addition, we established the PPI network of the DEGs and ten hub genes (*TGFB1*, *JUN*, *CXCL8*, *LRRK2*, *CD44*, *DCN*, *BCL2*, *ACACB*, *CCND1*, and *CXCL12*) were identified and verified by RT-PCR experiments in our own thyroid samples in Huashan Hospital. These findings may provide us with a better understanding of the molecular mechanism and possible treatment targets for PTC.

## 2. Materials and Methods

### 2.1. Gene Expression Profile Data

The gene expression profile datasets GSE33630, GSE35570, and GSE60542 were downloaded from GEO database. A platform of these three GEO datasets was GPL570 (Affymetrix Human Genome U133 Plus 2.0 Array). The GSE33630 dataset included 94 samples derived from PTC (papillary thyroid cancer) patients (49 tumor tissues and 45 nontumoral thyroid tissues). In the GSE35570 dataset, there consists of 32 PTC tissues and 51 normal samples. As for the GSE60542 dataset, 63 samples were included with 33 tumor tissues and 30 noncancerous tissues. We chose these three datasets for integrated analysis in this study because all of them had a pretty large sample size compared to other datasets about PTC in GEO data repository.

### 2.2. DEG Identification

A GEO2R (https://www.ncbi.nlm.nih.gov/geo/geo2r/) online program was applied to detect differentially expressed genes between PTC tumor and nontumor tissues [21]. ∣Log_2_FC∣ > 1 and the adjusted *p* value < 0.05 were set as cutoff criteria. Then, a total of 831 DEGs were identified, containing 410 upregulated genes and 421 downregulated genes, and we selected the top ten genes with highest degree of connectivity as hub genes. For the validation of the expression of these hub genes, the data from GEPIA was extracted and analyzed. The TCGA (The Cancer Genome Atlas) PTC data included 337 normal and 512 PTCs in the GEPIA. The GEPIA (http://gepia.cancer-pku.cn/) is a newly developed web-based tool to provide differential expression analysis about numerous kinds of cancer based on the data from TCGA and GTEx (Genotype-Tissue Expression) [22].

### 2.3. Functional Network Establishment of DEGs

To annotate the upregulated and downregulated DEGs presented from our analysis on the functional level, we discovered the gene ontology (GO) functional enrichment term including biological process (BP), cellular component (CC), molecular function (MF), and vital pathways using the online application DAVID (version 6.8, https://david.ncifcrf.gov/), Reactome (http://www.reactome.org), and clusterProfiler package in R (http://www.bioconductor.org/packages/release/bioc/html/clusterProfiler.html), with the threshold set as *p* < 0.05 [23].

### 2.4. PPI Network and Module Analysis

To determine the relationship between upregulated and downregulated DEGs, we uploaded all DEGs to the STRING database (version 10.5, https://string-db.org/cgi/input.pl), choosing a combined score > 0.4 to construct PPI. Then, the Cytoscape software (version 3.6.0, http://www.cytoscape.org/) was utilized to construct the network of PPI. The Molecular Complex Detection (MCODE) app in Cytoscape was recruited to analyze modules of the PPI network. The KEGG pathway analysis of genes in each module was performed by DAVID.

### 2.5. Tissue Samples

32 pairs of primary human PTC cancerous tissues and their corresponding adjacent normal thyroid tissues were obtained from Huashan Hospital of Fudan University. Samples were obtained and snap frozen at liquid nitrogen immediately and stored at -80°C until analysis. All PTC specimens were confirmed by histopathology. Ethical approval was obtained from the ethics committee of Huashan Hospital, Fudan University, and written informed consent was obtained from each patient.

### 2.6. RNA Extraction and RT-PCR

Total RNA was isolated from PTC cells and tissues using the TRIzol reagent (Invitrogen, USA). cDNA was synthesized using 2.0 *μ*g of total RNA with the PrimeScript™ RT reagent kit (TaKaRa Biotechnology, Japan). The SYBR-Green Supermix kit (TaKaRa) was employed to detect the relative mRNA expression in the ABI 7900 instrument (Applied Biosystems Inc.). The relative expression levels were determined by the 2^-*ΔΔ*Ct^ method and normalized to internal control GAPDH [24, 25]. All qPCR reactions were performed in triplicate. The primers used to explore mRNA expression of ten hub genes were shown in Table 1.

### 2.7. Statistical Analysis

The expression of related genes was presented as mean ± SD and analyzed using the paired *t*-test. All data were analyzed with GraphPad Prism 7.0, and *p* < 0.05 was considered as a significant difference.

## 3. Results

### 3.1. Screening of DEGs between PTC and Normal Thyroid Tissues

**Figure 1:**
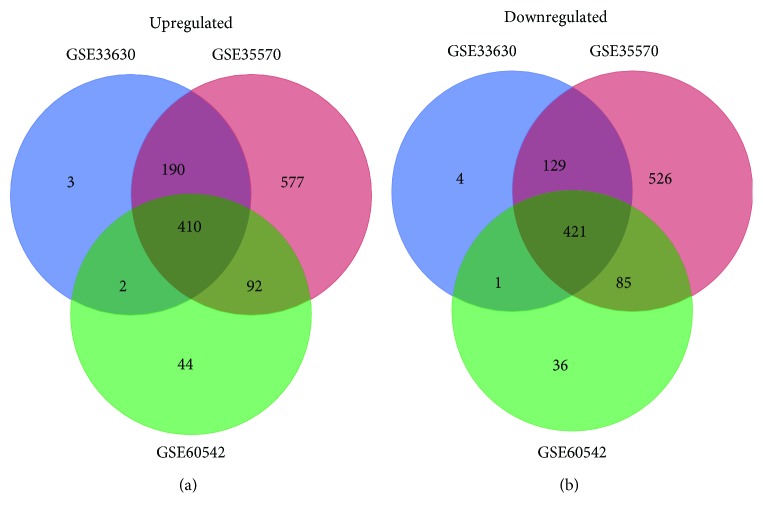
Identification of aberrantly expressed genes in gene expression profile datasets GSE33630, GSE35570, and GSE60542. Venn diagram of upregulated genes (a) and downregulated genes (b).

Three GEO datasets GSE33630, GSE35570, and GSE60542, a total of 114 PTC tissues and 126 normal thyroid tissues, were analyzed in our research. According to the cutoff criteria: ∣log_2_FC∣ > 1 and the adjusted *p* value < 0.05, we extracted 605, 1269, and 548 upregulated DEGs and 555, 1161, and 543 downregulated DEGs from the expression profile datasets GSE33630, GSE35570, and GSE60542, respectively (Figure 1). 410 upregulated DEGs and 421 downregulated DEGs were consistently presented in all three datasets and were used for further study.

### 3.2. Gene Ontology Functional Analysis

**Figure 2:**
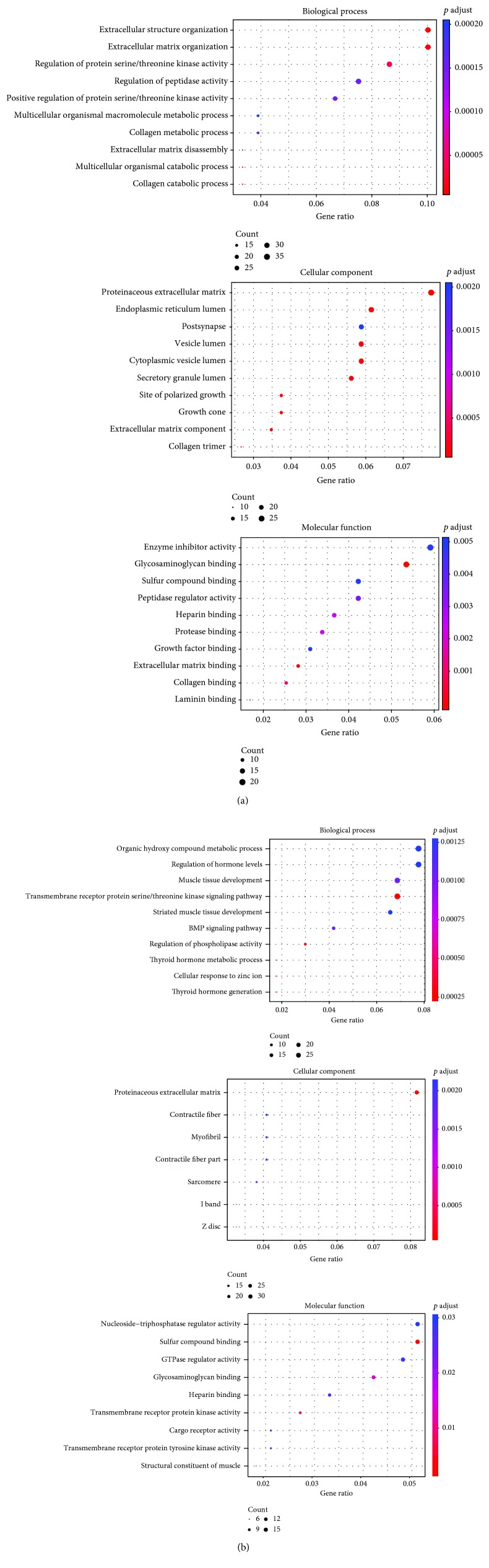
The enriched gene ontology categories including biological process, cellular component, and molecular function of DEGs. (a) The GO functional enrichment analysis of upregulated DEGs. (b) The GO functional enrichment analysis of downregulated DEGs based on clusterProfiler. Abbreviations: DEGs: differentially expressed genes; GO: gene ontology.

In order to obtain a more in-depth understanding of the selected DEGs, GO function was performed using DAVID version 6.8 and the clusterProfiler package in R statistical software (version 3.4.2). As shown in Table 2 and Figure 2(a), the upregulated DEGs mainly enriched in biological process (BP) terms were associated with extracellular matrix organization, cell adhesion, and regulation of protein serine/threonine kinase activity. Meanwhile, the downregulated DEGs were linked to nervous system development, muscle tissue development, and regulation of phospholipase activity (Figure 2(b)). As for the cellular component (CC), the upregulated DEGs were enriched in the proteinaceous extracellular region, endoplasmic reticulum lumen, and membrane-bounded vesicle, while the downregulated DEGs were related to the proteinaceous extracellular matrix. In the molecular function (MF) group, it was concluded that the upregulated DEGs were mainly involved in receptor binding and glycosaminoglycan binding and the downregulated DEGs were closely related to sulfur compound binding and nucleoside-triphosphatase regulator activity.

### 3.3. KEGG Signaling Pathway Enrichment Analysis

**Figure 3:**
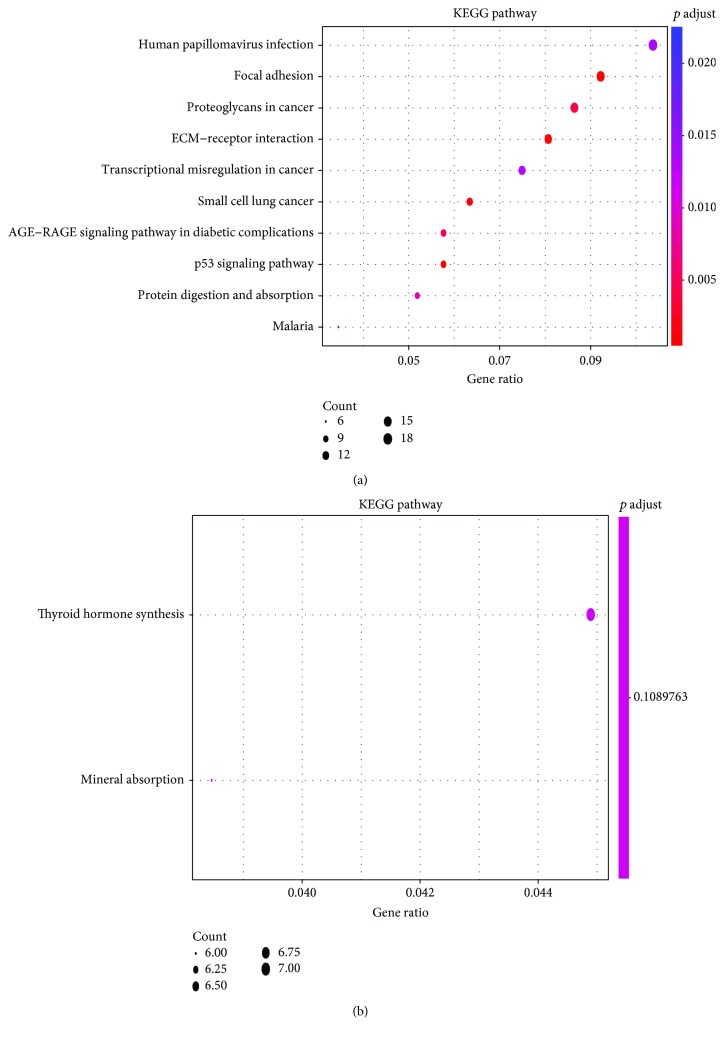
The results of KEGG pathways enrichment analysis for DEGs based on clusterProfiler: upregulated DEGs (a) and downregulated DEGs (b). Abbreviations: DEGs: differentially expressed genes; KEGG: Kyoto Encyclopedia of Genes and Genomes.

To systematically clarify the key pathways involved in PTC pathogenesis, pathway enrichment analysis was carried out using online websites of the KEGG pathway, Reactome, and the clusterProfiler R package. The pathways enriched by upregulated DEGs were mainly related to ECM-receptor interaction, focal adhesion, and p53 signaling pathway. However, the central pathways shown in downregulated DEGs were mineral absorption, thyroid hormone synthesis, and TGF-beta signaling pathway (Table 3 and Figure 3).

### 3.4. PPI Network Construction and Modular Analysis

**Figure 4:**
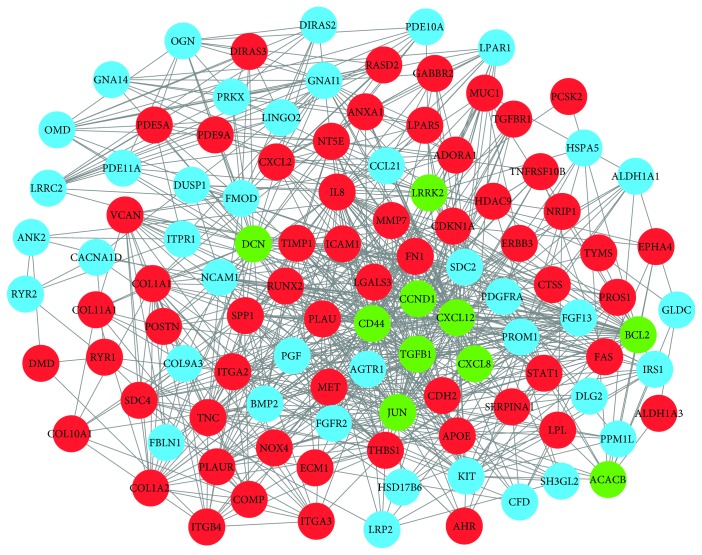
Protein-protein interaction network of differentially expressed genes. Red nodes represent upregulated genes. Blue nodes represent downregulated genes. Green nodes represent the top ten genes. Nodes > 10 was set as cutoff criteria.

**Figure 5:**
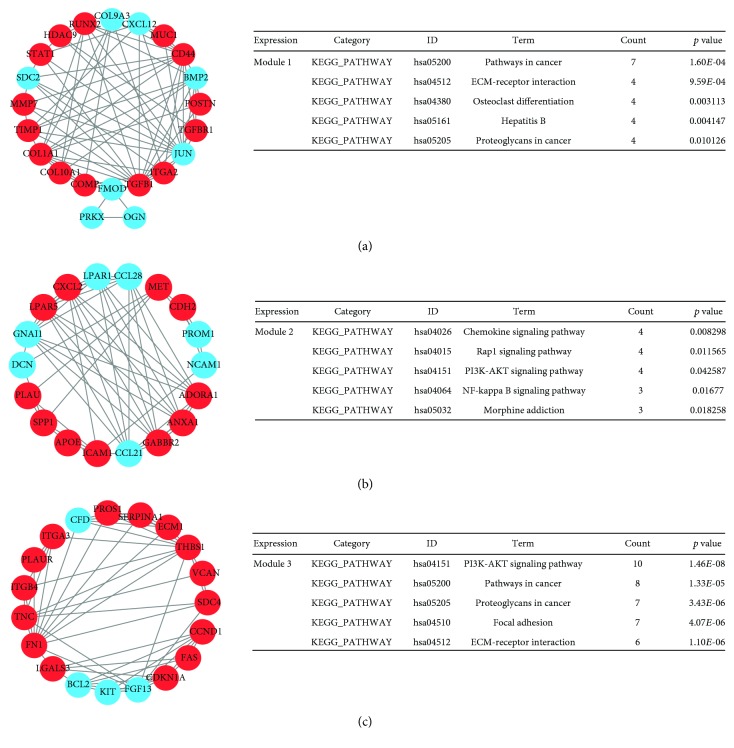
Top three modules with high degree of connectivity from the PPI network. (a) PPI Module 1 and KEGG pathway enrichment analysis for Module 1. (b) Module 2 and KEGG pathway analysis for Module 2. (c) Module 3 and KEGG pathway analysis for Module 3. Abbreviations: PPI: protein-protein interaction; KEGG: Kyoto Encyclopedia of Genes and Genomes.

The STRING online database was employed to construct the PPI network; then, the Cytoscape software was used to visualize the protein interactions. Considering degree > 10 as the cutoff criterion, a total of 108 DEGs (including 62 upregulated DEGs and 46 downregulated DEGs) in the 831 commonly altered DEGs were filtered to establish the PPI network, which comprises 108 nodes and 769 edges (Figure 4). The PPI network provides us with the most significant ten nodes (*TGFB1*, *CXCL8*, *LRRK2*, *CD44*, *CCND1*, *JUN*, *DCN*, *BCL2*, *ACACB*, and *CXCL12*), and they were chosen as the hub genes of PTC (Table 4). Furthermore, the whole PPI network was analyzed by MCODE based on the degree of importance. The top three modules were shown in Figure 5. Module 1 consists of 22 nodes and 71 edges, while Module 2 consists of 18 nodes and 54 edges, and there are 19 nodes and 54 edges in Module 3. Moreover, KEGG pathway enrichment analysis showed that genes in these three modules were predominantly associated with pathways in cancer, chemokine signaling pathway, and PI3K-Akt signaling pathway, respectively.

### 3.5. Validation of the DEGs by RT-PCR

**Figure 6:**
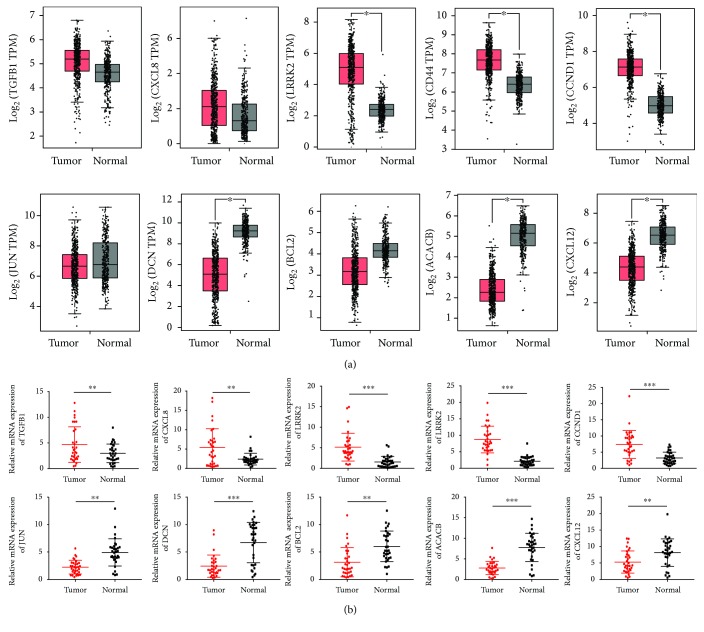
The expression level of *TGFB1*, *CXCL8*, *LRRK2*, *CD44*, *CCND1*, *JUN*, *DCN*, *BCL2*, *ACACB*, and *CXCL12*. (a) Validation of upregulated and downregulated hub genes based on TCGA and GTEx data in GEPIA. (b) Validation of the expression level of ten hub genes using RT-PCR. ^∗^*p* < 0.05, ^∗∗^*p* < 0.01, and ^∗∗∗^*p* < 0.001. Abbreviations: TCGA: The Cancer Genome Atlas; GTEx: Genotype-Tissue Expression; GEPIA: Gene Expression Profiling Interactive Analysis.

**Figure 7:**
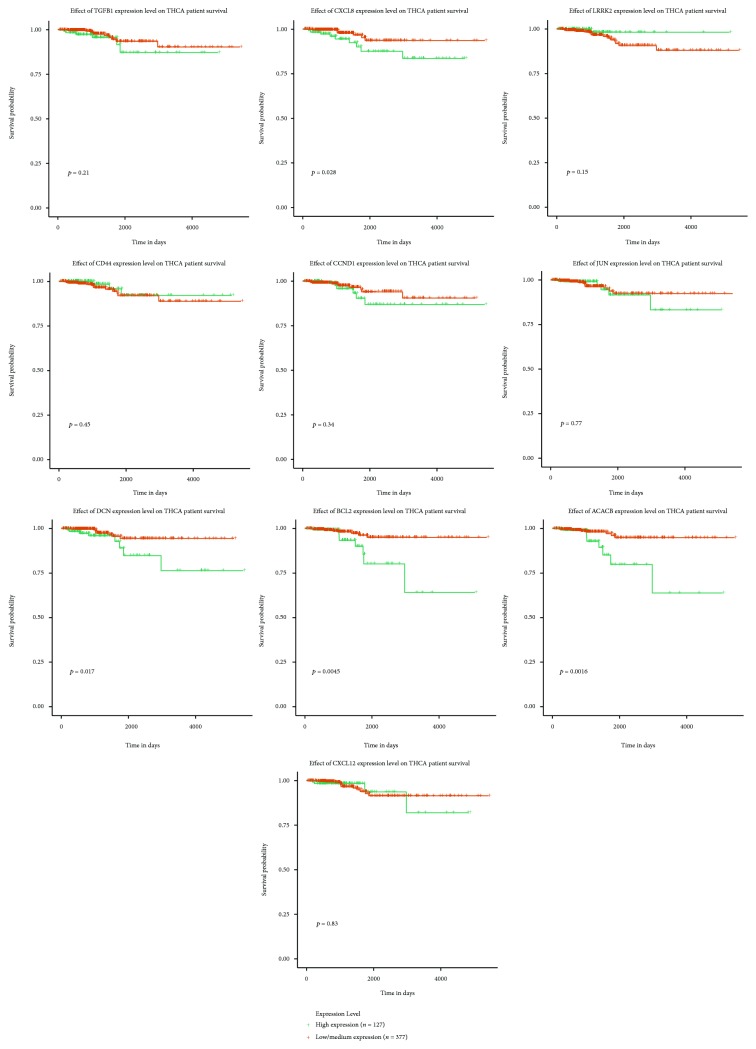
The Kaplan-Meier survival curve for 504 PTC patients with high and low expression of ten hub genes using data from TCGA database. Abbreviation: THCA: thyroid cancer.

To reinforce the reliability of the identified hub genes from the bioinformatics analysis, we firstly investigated the expression of these crucial genes in online website GEPIA based on TCGA and GTEx thyroid carcinoma (THCA) data. The results from TCGA and the GTEx projects containing 512 PTC samples and 337 normal samples showed that *TGFB1*, *CXCL8*, *LRRK2*, *CD44*, and *CCND1* were all elevated and *JUN*, *DCN*, *BCL2*, *ACACB*, and *CXCL12* were reduced to some extent in PTC tissues relative to normal tissues (Figure 6(a)). Moreover, RT-PCR experiment was conducted to reconfirm these hub genes. 32 pairs of PTC tissues and their adjacent noncancerous tissues collected in our cohort were used to measure the expression of these molecules. As indicated in Figure 6(b), the results of RT-PCR showed that the mRNA level of *TGFB1*, *CXCL8*, *LRRK2*, *CD44*, and *CCND1* increased by 2.44- (*p* = 0.0066), 3.64- (*p* = 0.0014), 6.67- (*p* < 0.0001), 5.58- (*p* < 0.0001), and 3.43-fold (*p* < 0.0001) and the level of *JUN*, *DCN*, *BCL2*, *ACACB*, and *CXCL12* decreased by 2.99- (*p* = 0.0024), 4.53- (*p* < 0.0001), 3.57- (*p* < 0.0035), 3.89- (*p* < 0.0001), and 2.37-fold (*p* = 0.0015), correspondingly. Although the fold changes varied from the data in GEO datasets and TCGA database, the total tendency of these genes from our experiment results was consistent with the databases. Besides, we evaluated the relationship between these hub genes and PTC patients' prognosis with UALCAN [26]. As shown in Figure 7, Kaplan-Meier survival curves revealed that PTC patients with high expression levels of *CXCL8* (*p* = 0.028), *DCN* (*p* = 0.017), *BCL2* (*p* = 0.0045), and *ACACB* (*p* = 0.0016) were correlated with a poor overall survival rate, suggested that these four molecules may serve as the prognostic markers for PTC. In brief, our study demonstrated that these ten hub genes may strongly link to the occurrence, progression, and prognosis of PTC and serve as a solid foundation for the future genomic individualized treatment of PTC.

## 4. Discussion

PTC is one of the most common endocrine-related cancers in the world, and the incidence increases steadily in recent decades [4, 27]. A previous study revealed that genetic alternations such as TERT mutations, BRAF V600E mutations, and RET/PTC rearrangement all contributed to the tumor proliferation and metastasis [28]. The precise mechanism concerning the pathogenesis of PTC remains largely elusive. Therefore, it is of great importance to identify and characterize the pivotal genes associated with the development of PTC. In the current study, our results provide a comprehensive bioinformatics analysis of genes and pathways which may participated in the progression of PTC. We integrated 3 datasets including 114 PTC tissues and 126 normal tissues, which enrolled the largest number of PTC tissue samples in similar bioinformatics studies, and identified 831 DEGs (410 upregulated and 421 downregulated) at the first step [29]. The following GO analysis showed that upregulated DEGs were mainly enriched in the extracellular matrix organization, endoplasmic reticulum lumen, and glycosaminoglycan binding. In addition, downregulated DEGs were significantly involved in the organic hydroxy compound metabolic process, proteinaceous extracellular matrix, and sulfur compound binding. Subsequent KEGG pathway enrichment analysis revealed that PTC development was strongly linked to pathways, such as thyroid hormone synthesis, ECM-receptor interaction, and p53 signaling pathway. In addition, we constructed the PPI network for DEGs and discovered top ten hub genes (*TGFB1*, *CXCL8*, *LRRK2*, *CD44*, *CCND1*, *JUN*, *DCN*, *BCL2*, *ACACB*, and *CXCL12*) with highest degree of connectivity. Finally, the most significant 3 modules were filter from the PPI network. Corresponding genes among these modules were associated with pathways in cancer, chemokine signaling pathway, and PI3K-Akt signaling pathway. Furthermore, experimental validation is indispensable to confirm our results predicted by bioinformatics analysis. RT-PCR results verified the overexpression of *TGFB1*, *CXCL8*, *LRRK2*, *CD44*, and *CCND1* and the downregulation of *JUN*, *DCN*, *BCL2*, *ACACB*, and *CXCL12* in 32 pairs of PTC samples and their adjacent normal tissues. Among these hub genes, *CXCL8*, *DCN*, *BCL2*, and *ACACB* were identified as clinically relevant genes using the expression status from the TCGA cohort of 504 PTC samples.

*CXCL8*, also named IL-8, is a member of the CXC chemokine family and produced by leukocytes and other cells. As a proinflammatory/chemoattractant cytokine, *CXCL8* exerts its oncogenic effect by sustaining cell proliferation, metastasis, and angiogenesis [30]. The results of our study were in consistent with several published research papers that have reported the elevated expression of *CXCL8* in PTC. Basolo et al. revealed that *CXCL8* was produced by TPC-1 and TT cells with activated RET/PTC1 arrangement and its expression was regulated by multiple intracellular signaling pathways [31]. Liotti et al. reported that the *CXCL8* expression level was correlated with lymph node metastases and it was overexpressed in thyrospheres and critical for self-renewal ability of thyroid cancer cells [32]. Rotondi et al. discovered that CXCL8 was highly abundant in PTC-derived tumor-associated macrophages (TAMs) and promoted PTC invasion in vivo [33]. Moreover, the role of CXCL8 in thyroid cancer was recently addressed by several studies which characterized its secretion and expression; in particular, these findings were recently reviewed and CXCL8 was proposed as the chemokine playing crucial protumorigenic effects in the cancer microenvironment [34]. Due to the important position of CXCL8 in PTC, Coperchini et al. designed some experiments to test the effect of the BRAF inhibitor (PLX4720) on the basal and TNF-*α*-induced CXCL8 secretions in BRAFV600E-mutated (BCPAP, 8305C, and 8505C) and RET/PTC-rearranged (TPC-1) thyroid cancer cell lines and in normal human thyrocytes (NHT). The experiment results show that PLX4720 is able to inhibit the secretion of CXCL8 in BRAFV600E-mutated thyroid cancer cells, which indicated that at least some of the antitumor activities of PLX4720 could be exerted through lowering of CXCL8 in the thyroid cancer microenvironment [35, 36].

Decorin (*DCN*) was known as an extracellular protein and a pan-RTK inhibitor, which participates in cancer cell growth, spread, proinflammatory processes, and antifibrillogenesis [37]. *DCN* was found underexpressed in breast cancer, colon cancer, follicular thyroid cancer, and follicular variant of papillary thyroid carcinomas [38, 39]. Mechanically, *DCN* binds to EGFR/ErB2 extracellular functional domains and triggers mitogen-activated protein kinase activation and the increase of cytosolic Ca^2+^, which accelerates cell cycle arrest and induces intrinsic cell apoptosis, eventually leading to tumor cell growth inhibition [40]. Taken together, the above studies highlighted *DCN* as a candidate anticancer target for many types of solid tumors. In addition, we have reason to suspect that DCN have an important role in the carcinogenesis of papillary thyroid cancer.

Acetyl-CoA carboxylases (ACC), the rate-limiting enzymes involved in the de novo synthesis of fatty acids, encode two isoforms ACC1 and ACC2 (*ACACB*) [41]. AMPK directly inhibits ACC1 and ACC2 activity via phosphorylation of ACC1 at Ser117 and ACC2 at Ser222, resulting in the inhibition of fatty acid synthesis and accumulation of fatty acid *β*-oxidation [42, 43]. A previous study has demonstrated an upregulation of ACC1 in multiple human cancers, which may promote lipogenesis to provide the energy for cancer cell growth [44]. However, we firstly found that the expression of ACC2 was at a very low level in PTC tissues and the specific mechanism of ACC2 in PTC carcinogenesis remains unclear and needs further investigation.

*BCL2* is an antiapoptotic oncogene, functioning in the regulation of cell intrinsic apoptosis. There exists numerous *BCL2* family members, including the antiapoptotic proteins *BCL2* and BCL-xL and proapoptotic proteins Bax, Bak, Bid, Noxa, Puma, Bad, and others [45]. Once received cellular stress, the activation of these *BCL2* family members releases apoptogenic proteins from the mitochondria and initiates caspase activation that results in cell apoptosis [46]. Overexpression of prosurvival *BCL2* in multiple cancers, such as prostate, ovarian, and lung cancer, is a hallmark for tumorigenesis [47–49]. Nevertheless, it was significantly downregulated in colorectal cancer (CRC) and PTC [50, 51]. Wang et al. investigated the relationship between *BCL2* polymorphisms and PTC susceptibility and found that *BCL2* (-938 C>A) polymorphism showed a protective role in susceptibility to PTC [52]. Gupta et al. reported downregulated *BCL2* in anaplastic papillary carcinoma (ATC) as compared to well-differentiated thyroid cancer [53]. Besides, loss of *BCL2* was associated with loss of differentiation in thyroid cancer. Our findings suggested that aberrantly expressed *BCL2* may have an unknown novel function as a potential tumor suppressor in thyroid cancer progression.

Taken together, we conducted a comprehensive bioinformatics analysis of DEGs in PTC and demonstrated that ten hub genes as the biomarkers of PTC and all these molecules were validated by our RT-PCR experiments. Our findings provide novel insights into the role of potential oncogenes and tumor suppressor genes in PTC and suggest that these findings may have a great clinical significance. Understanding of these core genes in thyroid cancer development as well as the molecular mechanisms involved in this process should be consolidated in the future investigation. Meanwhile, targeting these genes, especially the clinical relevant genes (*CXCL8*, *DCN*, *BCL2*, and *ACACB*), may provide effective therapies for the treatment of PTC. However, there is still a long way to get these genes transferred to the clinic for stratifying patients, taken as diagnostic biomarkers and even in depth for immunotherapy or for the development for oncovaccine.

## Figures and Tables

**Table 1:** Sequences of primers for RT-PCR.

Gene	Primer sequences
*TGFB1*	Forward: 5′-CAATTCCTGGCGATACCTCAG-3′
	Reverse: 5′-GCACAACTCCGGTGACATCAA-3′

*JUN*	Forward: 5′-TCCAAGTGCCGAAAAAGGAAG-3′
	Reverse: 5′-CGAGTTCTGAGCTTTCAAGGT-3′

*CXCL8*	Forward: 5′-ACTGAGAGTGATTGAGAGTGGAC-3′
	Reverse: 5′-AACCCTCTGCACCCAGTTTTC-3′

*LRRK2*	Forward: 5′-ATGAGTGGCAATGTCAGGTGT-3′
	Reverse: 5′-AATGTAAGCCTATGGAGCAAACA-3′

*CD44*	Forward: 5′-CTGCCGCTTTGCAGGTGTA-3′
	Reverse: 5′-CATTGTGGGCAAGGTGCTATT-3′

*DCN*	Forward: 5′-ATGAAGGCCACTATCATCCTCC-3′
	Reverse: 5′-GTCGCGGTCATCAGGAACTT-3′

*Bcl-2*	Forward: 5′-GGTGGGGTCATGTGTGTGG-3′
	Reverse: 5′-CGGTTCAGGTACTCAGTCATCC-3′

*ACACB*	Forward: 5′-CAAGCCGATCACCAAGAGTAAA-3′
	Reverse: 5′-CCCTGAGTTATCAGAGGCTGG-3′

*CCND1*	Forward: 5′-CAATGACCCCGCACGATTTC-3′
	Reverse: 5′-CATGGAGGGCGGATTGGAA-3′

*CXCL12*	Forward: 5′-CCATGCCGATTCTTCGAAAG-3′
	Reverse: 5′-TTCAGCCGGGCTACAATCTG-3′

*GAPDH*	Forward: 5′-GCACCGTCAAGGCTGAGAAC-3′
	Reverse: 5′-TGGTGAAGACGCCAGTGGA-3′

**Table 2:** Gene ontology analysis of differentially expressed genes in PTC.

Expression	Category	ID	Term	Count	*p* value
Downregulated	GOTERM_BP_FAT	GO:0007167	Enzyme-linked receptor protein signaling pathway	47	3.40*E*-08
	GOTERM_BP_FAT	GO:0007399	Nervous system development	80	8.50*E*-08
	GOTERM_BP_FAT	GO:0061061	Muscle structure development	34	1.20*E*-07
	GOTERM_BP_FAT	GO:0010517	Regulation of phospholipase activity	11	4.20*E*-07
	GOTERM_BP_FAT	GO:0040007	Growth	43	8.80*E*-07
	GOTERM_CC_FAT	GO:0005578	Proteinaceous extracellular matrix	31	1.50*E*-10
	GOTERM_CC_FAT	GO:0031012	Extracellular matrix	36	2.20*E*-09
	GOTERM_CC_FAT	GO:0044421	Extracellular region part	124	1.80*E*-07
	GOTERM_CC_FAT	GO:0005576	Extracellular region	137	2.50*E*-06
	GOTERM_CC_FAT	GO:0030018	Z disc	12	4.00*E*-05
	GOTERM_MF_FAT	GO:1901681	Sulfur compound binding	17	1.90*E*-05
	GOTERM_MF_FAT	GO:0005539	Glycosaminoglycan binding	16	2.00*E*-05
	GOTERM_MF_FAT	GO:0008201	Heparin binding	13	9.20*E*-05
	GOTERM_MF_FAT	GO:0005102	Receptor binding	49	4.30*E*-04
	GOTERM_MF_FAT	GO:0019199	Transmembrane receptor protein kinase activity	8	1.30*E*-03

Upregulated	GOTERM_BP_FAT	GO:0030198	Extracellular matrix organization	35	2.10*E*-14
	GOTERM_BP_FAT	GO:0007155	Cell adhesion	85	2.90*E*-14
	GOTERM_BP_FAT	GO:2000026	Regulation of multicellular organismal development	78	6.70*E*-11
	GOTERM_BP_FAT	GO:0040011	Locomotion	70	6.10*E*-10
	GOTERM_BP_FAT	GO:0051094	Positive regulation of developmental process	56	8.80*E*-10
	GOTERM_CC_FAT	GO:0005576	Extracellular region	160	2.20*E*-10
	GOTERM_CC_FAT	GO:0044421	Extracellular region part	141	2.40*E*-10
	GOTERM_CC_FAT	GO:0031982	Membrane-bounded vesicle	115	3.80*E*-05
	GOTERM_CC_FAT	GO:0070062	Extracellular exosome	94	4.90*E*-05
	GOTERM_CC_FAT	GO:1903561	Extracellular vesicle	94	6.00*E*-05
	GOTERM_MF_FAT	GO:0005539	Glycosaminoglycan binding	20	7.60*E*-08
	GOTERM_MF_FAT	GO:0005102	Receptor binding	60	5.40*E*-07
	GOTERM_MF_FAT	GO:0050840	Extracellular matrix binding	10	1.30*E*-06
	GOTERM_MF_FAT	GO:0042802	Identical protein binding	54	6.00*E*-06
	GOTERM_MF_FAT	GO:0098772	Molecular function regulator	52	5.60*E*-05

Abbreviations: GO: gene ontology; BP: biological process; MF: molecular function; CC: cellular component.

**Table 3:** KEGG and Reactome pathway enrichment analysis of differentially expressed genes in PTC.

Expression	Category	ID	Term	Count	*p* value
Downregulated	KEGG_PATHWAY	hsa04978	Mineral absorption	6	2.90*E*-03
	KEGG_PATHWAY	hsa04918	Thyroid hormone synthesis	7	3.80*E*-03
	KEGG_PATHWAY	hsa04350	TGF-beta signaling pathway	6	3.50*E*-02
	KEGG_PATHWAY	hsa00350	Tyrosine metabolism	4	3.90*E*-02
	KEGG_PATHWAY	hsa04020	Calcium signaling pathway	9	3.90*E*-02

Upregulated	KEGG_PATHWAY	hsa04512	ECM-receptor interaction	15	1.10*E*-08
	KEGG_PATHWAY	hsa04510	Focal adhesion	17	2.50*E*-05
	KEGG_PATHWAY	hsa04115	p53 signaling pathway	9	1.60*E*-04
	KEGG_PATHWAY	hsa05205	Proteoglycans in cancer	15	2.50*E*-04
	KEGG_PATHWAY	hsa04151	PI3K-Akt signaling pathway	17	7.20*E*-03

Downregulated	REACTOME_PATHWAY	R-HSA-5661231	Metallothioneins bind metals	6	2.33*E*-05
	REACTOME_PATHWAY	R-HSA-5660526	Response to metal ions	6	1.03*E*-04
	REACTOME_PATHWAY	R-HSA-3560782	Diseases associated with glycosaminoglycan metabolism	7	0.001726
	REACTOME_PATHWAY	R-HSA-8851708	Signaling by FGFR2 IIIa TM	5	0.001887
	REACTOME_PATHWAY	R-HSA-2129379	Molecules associated with elastic fibres	6	0.002228

Upregulated	REACTOME_PATHWAY	R-HSA-1474244	Extracellular matrix organization	35	1.39*E*-08
	REACTOME_PATHWAY	R-HSA-3000171	Nonintegrin membrane-ECM interactions	14	6.32*E*-08
	REACTOME_PATHWAY	R-HSA-6785807	Interleukin-4 and 13 signaling	26	7.33*E*-08
	REACTOME_PATHWAY	R-HSA-216083	Integrin cell surface interactions	16	1.25*E*-07
	REACTOME_PATHWAY	R-HSA-3000170	Syndecan interactions	9	1.29*E*-06

Abbreviations: KEGG: Kyoto Encyclopedia of Genes and Genomes; PTC: papillary thyroid carcinoma.

**Table 4:** Top 10 genes with high node degrees in PPI network.

Node	Degree	GSE33630		GSE35570		GSE60542	
		Log_2_FC	Adj. *p*	Log_2_FC	Adj. *p*	Log_2_FC	Adj. *p*
*TGFB1*	73	1.62	3.06 × 10^‐15^	2.52	1.04 × 10^‐14^	1.46	4.23 × 10^‐10^
*JUN*	67	-1.38	9.36 × 10^‐11^	-1.55	1.11 × 10^‐5^	-1.69	1.87 × 10^‐9^
*CXCL8*	62	2.01	2.22 × 10^‐9^	1.88	2.84 × 10^‐7^	1.11	1.91 × 10^‐3^
*LRRK2*	59	4.26	2.30 × 10^‐25^	5.98	9.14 × 10^‐35^	3.59	5.99 × 10^‐14^
*CD44*	56	1.88	3.16 × 10^‐15^	1.54	7.26 × 10^‐12^	1.38	2.48 × 10^‐13^
*DCN*	47	-1.88	1.58 × 10^‐14^	-2.26	5.32 × 10^‐12^	-1.75	8.24 × 10^‐6^
*BCL2*	46	-1.72	2.79 × 10^‐22^	-1.78	1.02 × 10^‐27^	-1.88	6.27 × 10^‐14^
*ACACB*	42	-1.11	9.88 × 10^‐13^	-1.56	3.93 × 10^‐18^	-1.45	1.53 × 10^‐16^
*CCND1*	42	1.71	1.32 × 10^‐20^	1.51	5.97 × 10^‐17^	1.27	6.31 × 10^‐7^
*CXCL12*	40	-1.56	1.43 × 10^‐13^	-1.76	6.17 × 10^‐10^	-1.74	1.08 × 10^‐8^

Notes: The adj. *p* value was calculated using Student's *t*-test. Abbreviations: PPI, protein-protein interaction.

## Data Availability

The data used to support the findings of this study are included within the article.
